# Supplementary material for: Efficacy of S-1 or Capecitabine Plus Oxaliplatin Adjuvant Chemotherapy for Stage II or III Gastric Cancer after Curative Gastrectomy: A Systematic Review and Meta-Analysis
Source: Cancers (Basel). 2022 Aug 16;14(16):3940. doi: 10.3390/cancers14163940 (PMC9406447; doi:10.3390/cancers14163940)
Supplement: Supplementary file 1 [file cancers-14-03940-s001.zip › cancers-1847499-supplementary.pdf]

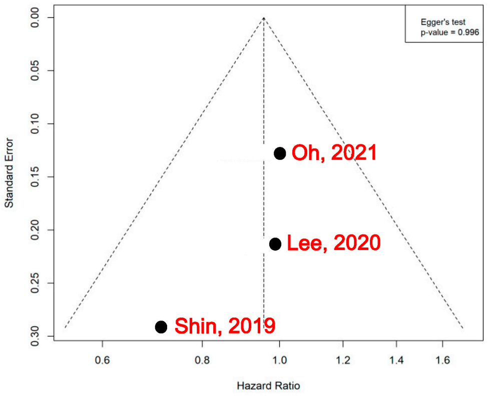

A. 5-year OS in all stages (II & III)

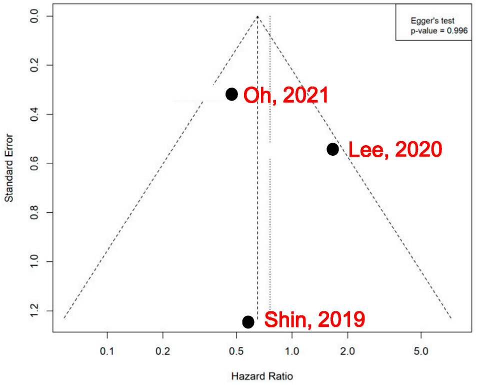

B. 5-year OS in stage II

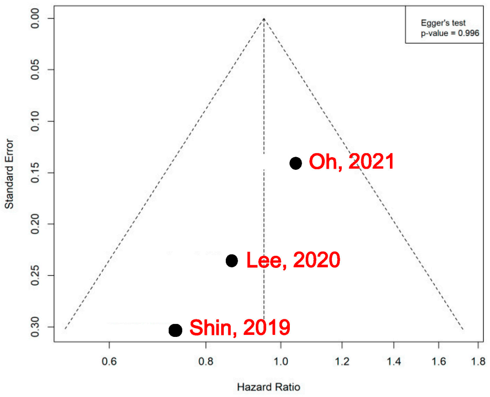

C. 5-year OS in stage III

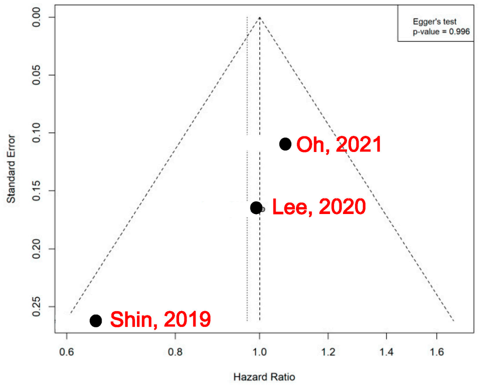

D. 5-year DFS in all stages (II & III)

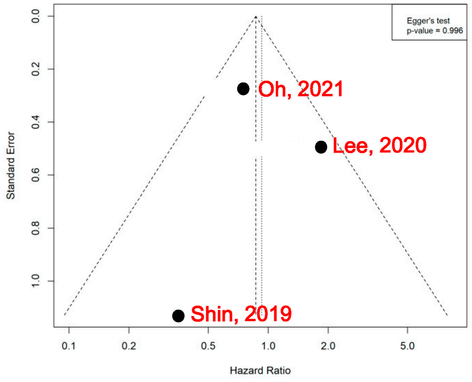

E. 5-year DFS in stage II

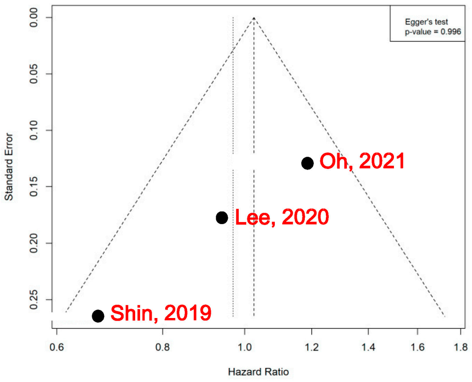

F. 5-year DFS in stage III

Supplementary Figure S1. Publication bias analyses of studies comparing S-1 and CAPOX regimens as adjuvant chemotherapy in stage II or III gastric cancers. Shin et al. [15]; Lee et al. [16]; Oh et al. [17].

Supplementary Table S1. Quality score of the three selected studies.

**1st author**	**Year of publication**	**Type of study**	**Group selection**	**Comparability between groups**	**Ascertainment of either the exposure or outcome of interest**	**Total score**
Shin et al. [15]	2019	Retrospective Cohort with PSM	3	2	3	8
Lee et al. [16]	2020	Retrospective Cohort with PSM	3	2	3	8
Oh et al. [17]	2021	Retrospective Cohort	3	1	3	7

PSM, Propensity Score Matching
